# Efficacy and safety of combined treatment of miniscalpel acupuncture and non-steroidal anti-inflammatory drugs: an assessor-blinded randomized controlled pilot study

**DOI:** 10.1186/s13063-017-2418-1

**Published:** 2018-01-12

**Authors:** Seungah Jun, Jung Hee Lee, Han Mi Gong, Yeon-Joong Chung, Ju-Ran Kim, Chung A. Park, Seong Hun Choi, Geon-Mok Lee, Hyun-Jong Lee, Jae Soo Kim

**Affiliations:** 1 Department of Acupuncture & Moxibustion medicine, College of Korean medicine, Daegu Haany University, 136, Sincheondong-ro, Suseong-gu, Daegu 706-828 Republic of Korea; 2 Department of Diagnostics, College of Korean medicine, Daegu Haany University, 136, Sincheondong-ro, Suseong-gu, Daegu 706-828 Republic of Korea; 3 Department of Anatomy and Histology, College of Korean medicine, Daegu Haany University, 136, Sincheondong-ro, Suseong-gu, Daegu 706-828 Republic of Korea; 4 Department of Acupotomy, LeeGeonmok Wonli Korean Medicine Hospital, Seoul, 137-829 Republic of Korea; 5 Department of Acupuncture & Moxibustion medicine, Daegu Oriental hospital of Daegu Haany University, 136, Sincheondong-ro, Suseong-gu, Daegu 706-828 Republic of Korea

**Keywords:** Miniscalpel acupuncture, Chronic neck pain, Pilot study, Non-steroidal anti-inflammatory drugs

## Abstract

**Background:**

Chronic neck pain is a common musculoskeletal disease during the lifespan of an individual. With an increase in dependence on computer technology, the prevalence of chronic neck pain is expected to rise and this can lead to socioeconomic problems. We have designed the current pilot study to evaluate the efficacy and safety of miniscalpel acupuncture treatment combined with non-steroidal anti-inflammatory drugs (NSAIDs) in patients with chronic neck pain.

**Methods:**

This seven-week clinical trial has been designed as an assessor-blinded, randomized controlled trial with three parallel arms. Thirty-six patients will be recruited and randomly allocated to three treatment groups: miniscalpel acupuncture treatment; NSAIDs; and miniscalpel acupuncture treatment combined with NSAIDs. Patients in the miniscalpel acupuncture and combined treatment groups will receive three sessions of miniscalpel acupuncture over a three-week period. Patients in the NSAIDs and combined treatment groups will receive zaltoprofen (one oral tablet, three times a day for three weeks). Primary and secondary outcomes will be measured at weeks 0 (baseline), 1, 2, 3 (primary end point), and 7 (four weeks after treatment completion) using the visual analogue scale and the Neck Disability Index, EuroQol 5-dimension questionnaire, and Patients’ Global Impression of Change scale, respectively. Adverse events will also be recorded.

**Discussion:**

This pilot study will provide a basic foundation for a future large-scale trial as well as information about the feasibility of miniscalpel acupuncture treatment combined with NSAIDs for chronic neck pain.

**Trial registration:**

Korean Clinical Research Information Service registry, KCT0002258. Registered on 9 March 2017.

**Electronic supplementary material:**

The online version of this article (doi:10.1186/s13063-017-2418-1) contains supplementary material, which is available to authorized users.

## Background

Chronic neck pain (CNP) is a common musculoskeletal disease with a lifetime prevalence of up to 67% [1]. According to a previous study, the one-year prevalence of neck pain was 29% and 40% for men and women, respectively [2]. In Korea, the lifetime prevalence of CNP is 20.8% [3]. With an increase in dependence on computer and smart phone technology, the prevalence rate of CNP will continue to rise. Pain can be directly related to substantial medicine consumption, absenteeism from work, unemployment, and disability [4, 5]. Therefore, symptom relief is important to improve the quality of life.

Medication, massage, exercise, physiotherapy, and patient education are common methods for the management of CNP [6]. However, the effects of these conventional therapies are limited and the validity of many CNP treatment guidelines is unclear [6, 7]. Although pain-control drugs such as non-steroidal anti-inflammatory drugs (NSAIDs) are one of the most commonly used drugs for CNP, their long-term use carries a risk of side effects and intolerance [8]. Modalities from the field of complementary and alternative medicine (CAM) are also frequently used for treatment [9]. These include acupuncture, herbal medicine, cupping, moxibustion, and pharmacopuncture [10]. Patients with chronic musculoskeletal disease tend to utilize both conventional and CAM treatments for more positive outcomes [11].

Miniscalpel acupuncture (MA) tends to exert better therapeutic effects on chronic musculoskeletal pain compared with regular acupuncture treatment [12]. MA combines Traditional Chinese Medicine meridian theory and modern surgical principles and it requires an understanding of anatomical structures. The aim of MA is recovery of the kinetic state of soft tissue through peeling adhesion and removal of attached tissues [13], with the advantage of quicker recovery and pain reduction compared with regular acupuncture [14]. Furthermore, it shows superior and long-lasting effects compared with regular acupuncture [15].

Although CAM and conventional treatment modalities are frequently applied in combination, their efficacy and safety have rarely been investigated in clinical trials. In this pilot study, we will investigate the efficacy and safety of MA combined with NSAIDs in patients with CNP. The results will provide clinical evidence for evaluating the feasibility of large-scale randomized controlled trials (RCTs) of this combination treatment for CNP.

## Methods/Design

### Design

The protocol of this assessor-blinded RCT follows the Declaration of Helsinki and Korean Good Clinical Practice. The study will be conducted at Daegu Oriental Hospital of Daegu Haany University, Daegu, Republic of Korea. Independent researchers blinded to randomization will assess the outcomes and analyze statistics. Informed consent to participate in the clinical trial will be obtained after the researcher provides sufficient information and explanations regarding the study interventions. This protocol follows the SPIRIT guidelines and the checklist and figure are available at the end of the manuscript.

This study is designed as a pilot trial to calculate the appropriate sample size for further RCTs. Each group will include 12 participants, allowing for a 20% dropout rate for evaluating the pragmatic purpose of this trial. A total number of 36 participants will be included, which is larger than the minimum number recommended for pilot studies (Additional files 1 and 2).

### Participants

**Fig. 1:**
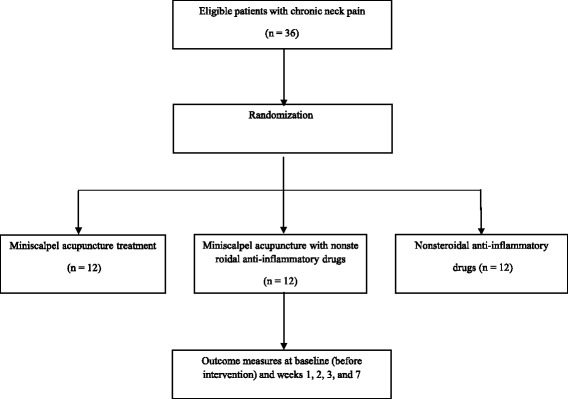
*Flow chart* of our pilot randomized controlled trial

One of the main objectives of this study is to provide an estimate of the sample size required for the full-scale clinical RCT. In total, 36 participants with CNP will be recruited through advertisements in local newspapers, on hospital websites, and on bulletin boards. If individuals are interested in participating, they will be invited to the hospital for a screening visit. Eligibility will be determined by one researcher on the basis of the results of physical and radiographic assessments. After completing the screening questionnaire, participants will be guided through the informed consent process. They will be informed about randomization to any one of three treatment groups: MA; NSAIDs; and combined treatment groups. Written informed consent will be obtained from each participant before the initiation of any treatment. After consent forms are obtained, participants will be randomly allocated to one of the three groups in a 1:1:1 ratio (Fig. 1).

### Eligibility criteria

Individuals aged 19–75 years who agree to participate after providing written informed consent will be selected as study volunteers. The inclusion criteria are as follows: men or women aged 19–75 years; symptoms such as neck pain and stiffness lasting for ≥ three months; a baseline score of ≥ 4 on the visual analog scale (VAS) for pain; and provision of written informed consent.

Exclusion criteria are as follows: individuals with abnormalities in neurological functions such as cervical nerve function, deep tendon reflexes, and muscle and sensory functions; those with serious sensory or motor disorders requiring surgical treatment; those with a history of spinal surgery or those with procedures scheduled during the study; those with spinal disorders, including vertebral fracture, spinal infection, inflammatory spondylitis, and malignancy, on computed tomography or magnetic resonance imaging; those who are pregnant, planning pregnancy, breast feeding, or refusing to consume certain forms of contraceptives; those with other chronic diseases that can affect the outcomes of the study interventions, including cardiovascular disease, diabetic neuropathy, active hepatitis, fibromyalgia, rheumatoid arthritis, dementia, and epilepsy; those with other severe musculoskeletal problems in areas other than the neck; those with serious psychiatric or psychological disorders; those using aspirin or anticoagulant medications; those using NSAIDs; those with abnormal findings in blood tests for renal or hepatic function; and those deemed ineligible by the recruiting researcher.

### Randomization and allocation concealment

The 36 patients will be randomized using simple randomized procedures, with a 1:1:1 allocation ratio. Written informed consent will be obtained before randomization. Random numbers will be generated by an independent statistics professional using SPSS version 19.0 for Windows (release 19.0 K; SPSS Inc. Armonk, NY, USA). Sealed opaque assignment envelopes will be used for allocation concealment. The participants will be allocated into the predefined treatment arms. Allocation concealment will not be broken until study completion. The treatment arm and random number of an individual participant will be recorded in the case report form and randomization table. Outcome assessors and data analysts will be blinded to interventions.

### Interventions

The MA group will receive MA three times over three weeks. The NSAIDs group will be prescribed zaltoprofen 80 mg/Tab (Soleton, CJ Health Care Co., Ltd, Seoul, Republic of Korea) at a dose of one tablet administered orally three times per day over three weeks. The combined treatment group will receive MA and Soleton over three weeks. The prescription will be administered in every visit and drugs that have not been taken will be withdrawn. During the study period, all other interventions such as moxibustion and physical therapy, surgical procedures, and injections for neck pain will be prohibited. Other medications that do not influence neck pain will be allowed. Any change will be recorded at every visit.

### MA

**Fig. 2:**
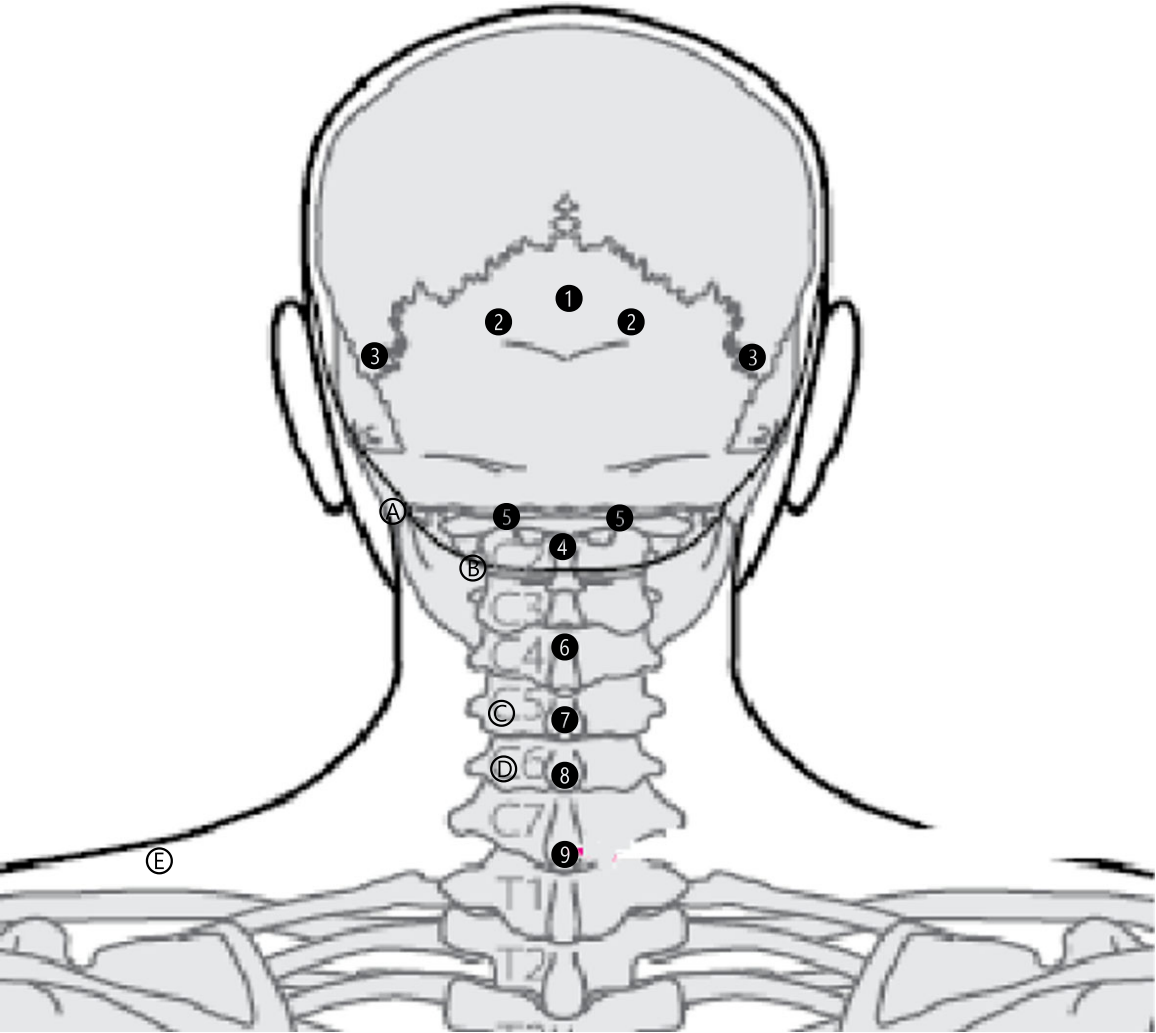
Miniscalpel acupuncture points: (1) below external occipital protuberance, insertion of nuchal ligament, origin of trapezius muscle, upper site of GV16; (2) 2–25.cm side down of external occipital protuberance, rectus capitis posterior major, obliquus capitis superior, GB20; (3) 4–4.5 cm side down of external occipital protuberance, GB12; (4) C2 spinous process, origin of semispinalis capitis, obliquus capitis inferior, rectus capitis posterior major, GV12; (5) side of C2 spinous process, facet joint, BL10; (6) C4 spinous process, origin of semispinalis capitis; (7) C5 spinous process, origin of semispinalis capitis; (8) C6 spinous process, origin of semispinalis capitis; (9) C7 spinous process, GV14; (**a**) transverse process of atlas, origin of levator scapulae, obliquus capitis superior, obliquus capitis inferior; (**b**) transverse process of axis, origin of levator scapulae, medial scalene; (c) lamina of C5 (2 cm side of C5 spinous process), splenius capitis, semispinalis capitis; (d) lamina of C6 (2 cm side of C6 spinous process), splenius capitis, semispinalis capitis; (e) GB21, upper trapezius

The MA points will be as follows [16]: upper site of GV16 (insertion of nuchal ligament, origin of trapezius muscle); GB20; GB12; GV15; BL10; GV14; C4 spinous process (origin of semispinalis capitis); C5 spinous process (origin of semispinalis capitis); and C6 spinous process (origin of semispinalis capitis). A total of 12 sites that appeal to pain will be selected for treatment. The sites can be increased to a maximum of 20 according to the participant’s condition: transverse process of C1 (origin of levator scapulae); transverse process of C2 (origin of levator scapulae and medial scalene); lamina of C5 (2 cm side of spinous process); lamina of C6 (2 cm side of spinous process); and GB21 (Fig. 2). A sterilized, disposable miniscalpel (DongBang Acupuncture Inc., Republic of Korea; 0.5 × 50 mm) will be used. MA will be performed by Korean medical doctors (KMD) licensed by the Ministry of Health and Welfare. The KMD will wear a sterile mask and surgical gloves. After sterile skin preparation, participants will be administered MA at a depth of tendon or ligament level. After treatment completion, the KMD will check for any abnormality and bleeding.

### Outcomes

The primary outcome will be measured using VAS. Changes in VAS scores after three weeks of treatment will be measured relative to the baseline scores and will be considered the primary outcome. The secondary outcome will be determined by the Neck Disability Index (NDI), EuroQol 5-dimension (EQ-5D) questionnaire, and Patient Global Impression of Change (PGIC) scale. Outcomes will be measured at weeks 0 (baseline), 1, 2, 3 (primary end point), and 7 (four weeks after treatment completion). Outcome measurements will be recorded by two independent researchers. Table 1 shows the treatment and outcome measurement schedules.

**Table 1:** Treatment and outcome measurement schedules during our pilot study

Outcome measures	Screening	Week 0 (baseline)	Week 1	Week 2	Week 3	Week 7
Screening test	V					
Sociodemographic characteristics	V					
Randomization		V				
Laboratory test	V				V	
MA treatment		V	V	V		
NSAIDs prescription		V	V	V		
Visual analog scale	V	V	V	V	V	V
Neck Disability Index		V	V	V	V	V
EQ-5D questionnaire		V	V	V	V	V
PGIC scale			V	V	V	V
Adverse events		V	V	V	V	V
Credibility assessment					V	
Total safety assessment					V	

*NSAIDs* nonsteroidal anti-inflammatory drugs, *EQ-5D* EuroQol 5-dimension, *PGIC* Patient’s Global Impression of Change

### Primary outcome measurement

VAS is a 10-cm measurement instrument to determine the severity of pain. A 10-cm horizontal line will be used. The individuals rate his or her pain on a scale of 0–10, where 0 indicates the absence of pain and 10 indicates the worst pain imaginable. Participants will estimate their level of pain by marking on the line. Then the distance from “absence of pain” point will be measured [17, 18]. In this study, the VAS score will indicate pain at the point of measurement. Changes in VAS scores after three weeks of treatment, relative to the baseline scores, will be considered the primary outcome.

### Secondary outcome measurement

#### VAS for pain intensity

Pain intensity will be assessed using a 10-cm VAS. Participants will be asked to report the intensity of pain at weeks 0 (baseline), 1, 2, 3, and 7.

#### NDI

Neck pain-related disability will be evaluated using the validated Korean version of NDI. NDI is used to measure self-perceived disability caused by neck pain. It comprises ten items for pain intensity, concentration, functional activities, and headache [19]. Each question is assessed using a 6-point scale of 0–5, where 0 indicates the absence of a problem and 5 indicates severe disability caused by neck pain.

#### EQ-5D

The Korean version of the EQ-5D questionnaire will be used to measure the health-related quality of life of our patients with CNP. This questionnaire comprises five dimensions, including mobility, self-care, usual activities, pain/discomfort, and anxiety/depression [20]. Each dimension is rated on a scale of 1–3, where 1 indicates no problem and 3 indicates a major problem.

#### PGIC

At the end of each treatment session, the participants will complete the PGIC scale, which is a 7-point categorical scale. They will be asked to describe changes in activity limitations, symptoms, emotions, and overall quality of life. The PGIC scale is one of the most commonly used method of assessing clinically meaningful changes that make a difference to patients in studies on musculoskeletal conditions [21].

### Safety

The safety of this trial will be assessed by the red blood cell count, hemoglobin level, hematocrit, total white blood cell count, differential count, erythrocyte sedimentation rate, platelet count, aspartate aminotransferase level, alanine aminotransferase level, blood urea nitrogen level, prothrombin time, partial thromboplastin time, C-reactive protein and creatinine levels, serum sodium level, serum potassium level, and serum chloride level. All participants will undergo blood tests twice during the study, once at screening and once at the fourth follow-up visit. Nexina (potassium bismuth citrate 100 mg/Tab, ranitidine hydrochloride 84 mg/Tab, sucralfate hydrate 300 mg/Tab) will be prescribed if the participants complain of dyspepsia symptoms such as nausea, abdominal discomfort, and diarrhea.

All adverse events (AEs) and vital signs will be observed at every visit. Serious AEs (SAEs) will be reported to the IRB. The individuals will be asked to voluntarily report information about AEs and the researchers will confirm the occurrence of AEs through methods such as medical interviews. Details about AEs, such as the date of occurrence, degree of severity, causal relationship with the treatment, other treatments or medications that are suspected to cause the AE, and treatment of the AE, will be reported in detail.

### Withdrawal and dropout

All participants will have the right to withdraw from the study at any time. Participation will be terminated at any stage if the individual refuses to continue, withdraws consent, or violates the inclusion or exclusion criteria. The trial will be stopped if the principal investigator believes that there are unacceptable risks of SAEs.

### Statistical analysis

The results of this study will provide a preliminary report regarding the efficacy and safety of MA treatment combined with NSAIDs for CNP. All statistical analyses will be performed using IBM SPSS version 19.0 for Windows (Release 19.0 K; IBM SPS Inc., Armonk, NY, USA) and will be based on the Statistics Guidelines for Clinical Trials [22]. The significance level of the tests will be set at 0.05. One-way analysis of variance (ANOVA) and descriptive analysis will be performed to compare baseline characteristics between groups. Trends over time and time-by-treatment interactions will be explored using repeated measures ANOVA. The Chi-square test or Fisher’s exact test will be performed to compare differences in ratios caused by AEs between groups.

### Data monitoring

A qualified clinical research associate will oversee study protocol compliance, informed consent documents, overall progress of the trial, participant recruitment, data quality and timeliness, performance of the interventions, and all fields and process of the trial. If any important protocol modifications or violations exist, an amended protocol will be resubmitted and reported to the relevant parties (e.g. investigators, IRB, trial participants, trial registries, and sponsor).

## Discussion

The current management of CNP involves a comprehensive approach including pharmacological and non-pharmacological interventions. Recently, there have been increased interests in the use of CAM modalities for the treatment of CNP. CAM modalities have recently been receiving more attention for pain control. Acupuncture is the one of the most widely known CAM modalities. In previous studies, acupuncture was proven effective for the treatment of CNP [23]. Of late, a variety of acupuncture treatments such as thread embedding therapy, MA, and pharmacopuncture are being practiced in Korea; these tend to exert better effects compared with traditional acupuncture [24, 25].

The use of MA is increasing in Korea. From January 1999 to May 2014, 28 clinical research papers on MA were documented in Korea [26], and this approach has come to be widely used for the treatment of musculoskeletal diseases [27]. In a review of trends regarding MA in Korea, 87.1% MA studies pertained to musculoskeletal disease and approximately 14.7% of the musculoskeletal diseases involved neck pain. MA is reportedly effective for the management of cervical herniated intervertebral discs and other cervical diseases such as ossification of the posterior longitudinal ligament [28, 29]. As stated above, previous studies have indicated positive results regarding MA treatment for neck pain, although clinical studies on combination treatment involving western medicine approaches such as the use of painkillers and MA are lacking.

The present study will be conducted by researchers trained to follow the study protocol and good clinical practice guidelines. Methodological integrity and scientific validity will be guaranteed with strict supervision and monitoring. The results of this study will contribute to a better understanding of the efficacy and safety of MA treatment alone and in combination with NSAIDs, which are frequently used for the treatment of CNP. Furthermore, they will provide guidelines for the development of a relevant protocol for conducting a RCT of MA treatment combined with NSAIDs for CNP and will aid researchers in calculating the effect size for such full-scale RCTs. Finally, they will provide evidence regarding the combination of Korean and western medicine modalities for the management of CNP.

### Trial status

This trial began in March 2017 after IRB approval. Trial completion is expected by the end of September 2017. Recruitment is currently ongoing.

## Additional files

Additional file 1: SPIRIT 2013 Checklist: Recommended items to address in a clinical trial protocol and related documents. (DOC 120 kb)

Additional file 2: Table S1. STRICTA (Standards for Reporting Interventions in Clinical Trials of Acupuncture) of experimental group interventions. (DOCX 16 kb)

## Abbreviations

ANOVA: Analysis of variance
CAM: Complementary and alternative medicine
CNP: Chronic neck pain
EQ-5D: Euroqol 5-dimension
IRB: Institutional review board
KMD: Korean medical doctors
MA: Miniscalpel acupuncture
NDI: Neck disability index
NSAIDs: Non-steroidal anti-inflammatory drugs
PGIC: Patient global impression of change
