# Supplementary material for: Efficacy and safety of combined treatment of miniscalpel acupuncture and non-steroidal anti-inflammatory drugs: an assessor-blinded randomized controlled pilot study
Source: Trials. 2018 Jan 12;19:36. doi: 10.1186/s13063-017-2418-1 (PMC5766990; doi:10.1186/s13063-017-2418-1)
Supplement: Supplementary file 2 — STRICTA (Standards for Reporting Interventions in Clinical Trials of Acupuncture) of experimental group interventions. (DOCX 16 kb) [file 13063_2017_2418_MOESM2_ESM.docx]

**Additional file 2: Table S1. STRICTA (Standards for Reporting Interventions in Clinical Trials of Acupuncture) of experimental group interventions**

| **Item** | **Detail** | **answer** |
| --- | --- | --- |
| **1. Acupuncture rationale** | 1a) Style of acupuncture (e.g. Traditional Chinese Medicine, Japanese, Korean, Western medical, Five Element, ear acupuncture, etc) | Miniscalpel acupuncture |
|  | 1b) Reasoning for treatment provided, based on historical context, literature sources, and/or consensus methods, with references where appropriate | Published articles* |
|  | 1c) Extent to which treatment was varied | 6 predefined acupuncture points and C4 spinous process, C5 spinous process, and C6 spinous process. |
| **2. Details of needling** | 2a) Number of needle insertions per subject per session (mean and range where relevant) | 12 needle insertions (increased to a maximum of 20 according to the participant’s condition) |
|  | 2b) Names (or location if no standard name) of points used (uni/bilateral) | GV16, GB20, GB12, GV16, BL10, GV14, C4 spinous process, C5 spinous process, and C6 spinous process |
|  | 2c) Depth of insertion, based on a specified unit of measurement, or on a particular tissue level | The sterilized disposable MA (DongBang Acupuncture Inc., Korea) 0.5mm × 50m in size will be used. The needle is inserted into tendon or ligament level. |
|  | 2d) Response sought (e.g. *de qi* or muscle twitch response) | - |
|  | 2e) Needle stimulation (e.g. manual, electrical) | Miniscalpel acupuncture |
|  | 2f) Needle retention time | After invasion, no retention time needed |
|  | 2g) Needle type (diameter, length, and manufacturer or material) | Sterilized disposable acupuncture (Dongbang Acupuncture Inc., Korea) 0.5mm × 50mm |
| **3. Treatment regimen** | 3a) Number of treatment sessions | once per week |
|  | 3b) Frequency and duration of treatment sessions | three times per three weeks |
| **4. Other components of treatment** | 4a) Details of other interventions administered to the acupuncture group (e.g. moxibustion, cupping, herbs, exercises, lifestyle advice) | Not done |
|  | 4b) Setting and context of treatment, including instructions to practitioners, and information and explanations to patients | Written informed consent will be obtained |
| **5. Practitioner background** | 5) Description of participating acupuncturists (qualification or professional affiliation, years in acupuncture practice, other relevant experience) | Korean medical doctors who are licensed by Ministry of Health and Welfare |
| **6. Control or comparator interventions** | 6a) Rationale for the control or comparator in the context of the research question, with sources that justify this choice | Routine treatment** |
|  | 6b) Precise description of the control or comparator. If sham acupuncture or any other type of acupuncture-like control is used, provide details as for Items 1 to 3 above. | Non-steroidal anti-inflammatory drugs |

* Park S, Kim S, Kim J, Kim S, Lee G. The comparative study of effects between acupotomy and its cotreatment with spine decompression therapy on HIVD patients. The Acupuncture. 2012;29(3):29-39.

*Guo C, Liu N, Li X, Sun H, Hu B, Lu J, et al. Effect of acupotomy on nitric oxide synthase and beta-endorphin in third lumbar vertebrae transverse process syndrome model rats. Journal of Traditional Chinese Medicine. 2014;34(2):194-8.

*Ma C, Wu S, Li G, Xiao X, Mai M, Yan T. Comparison of miniscalpel-needle release, acupuncture needling, and stretching exercise to trigger point in myofascial pain syndrome. Clin J Pain. 2010;26(3):251-7.

*Lee G, Kim D, Kim H, Yeom S, Kim H, Kim D. The study on the effect of acupotomy in lumbar HIVD. The Journal of Korean Acupuncture & Moxibustion Medicine Society. 2008;25(4):183-90.

*Kim S. Clinical characteristics of poor responders to acupotomy and safety pretreatment management. The Acupuncture. 2008;25(4):117-25.

** Aker PD, Gross AR, Goldsmith CH and Peloso P. Conservative management of mechanical neck pain: systematic overview and meta-analysis. BMJ. 1996;313:1291-6
